# mPGES-1 in prostate cancer controls stemness and amplifies epidermal growth factor receptor-driven oncogenicity

**DOI:** 10.1530/ERC-15-0277

**Published:** 2015-08

**Authors:** Federica Finetti, Erika Terzuoli, Antonio Giachetti, Raffaella Santi, Donata Villari, Hiromi Hanaka, Olof Radmark, Marina Ziche, Sandra Donnini

**Affiliations:** 1 Department of Life Sciences, University of Siena, Via Aldo Moro 2, Siena, 53100, Italy; 2 Department of Surgery and Translational Medicine, University of Florence, Largo Brambilla 3, Firenze, 50134, Italy; 3 Department of Clinical and Experimental Medicine, University of Florence, Viale Pieraccini 18, Firenze, 50139, Italy; 4 Department of Medical Biochemistry and Biophysics, Karolinska Institutet, Stockholm, SE-171 77Sweden; 5 Istituto Toscano Tumori (ITT), Firenze, Italy

**Keywords:** mPGES-1, prostate cancer, stemness, EGFR, EMT

## Abstract

There is evidence that an inflammatory microenvironment is associated with the development and progression of prostate cancer (PCa), although the determinants of intrinsic inflammation in PCa cells are not completely understood. Here we investigated whether expression of intrinsic microsomal PGE synthase-1 (mPGES-1) enhanced aggressiveness of PCa cells and might be critical for epidermal growth factor receptor (EGFR)-mediated tumour progression. In PCa, overexpression of EGFR promotes metastatic invasion and correlates with a high Gleason score, while prostaglandin E_2_ (PGE_2_) has been reported to modulate oncogenic EGFR-driven oncogenicity. Immunohistochemical studies revealed that mPGES-1 in human prostate tissues is correlated with EGFR expression in advanced tumours. In DU145 and PC-3 cell lines expressing mPGES-1 (mPGES-1^SC^ cells), we demonstrate that silencing or ‘knock down’ of mPGES-1 (mPGES-1^KD^) or pharmacological inhibition by MF63 strongly attenuates overall oncogenic drive. Indeed, mPGES-1^SC^ cells express stem-cell-like features (high CD44, β1-integrin, Nanog and Oct4 and low CD24 and α6-integrin) as well as mesenchymal transition markers (high vimentin, high fibronectin, low E-cadherin). They also show increased capacity to survive irrespective of anchorage condition, and overexpress EGFR compared to mPGES-1^KD ^cells. mPGES-1 expression correlates with increased *in vivo* tumour growth and metastasis. Although EGFR inhibition reduces mPGES-1^SC^ and mPGES-1^KD ^cell xenograft tumour growth, we show that mPGES-1/PGE_2_ signalling sensitizes tumour cells to EGFR inhibitors. We propose mPGES-1 as a possible new marker of tumour aggressiveness in PCa.

## Introduction

Prostate cancer (PCa) is currently treated with androgen deprivation and chemotherapeutic agents. Resistance to chemotherapy, a mounting issue in clinical oncology due to its association with tumour recurrence, has hastened the search for new prognostic biomarkers and new therapeutic targets aimed at patient stratification in relation to treatment (Schrecengost & Knudsen 2013, Augello *et al*. 2014). Because ∼40% of PCas express epidermal growth factor receptor (EGFR), expression of which is correlated with tumour recurrence and high Gleason score, the receptor is assumed to be a potential molecular target for advanced PCa. However, clinical trials in PCa patients have shown limited efficacy of EGFR-targeted drugs (Canil *et al*. 2005, Hammarsten *et al*. 2007, Schlomm *et al*. 2007, Gravis *et al*. 2008, Nabhan *et al*. 2009). Multiple mechanisms, such as unrestrained expression of EGFR, emergence of oncogenic mutants (KRAS, BRAF and PIK3CA) and inactivation of the PTEN tumour suppressor gene, underlie resistance to these drugs. Acquired EGFR antagonist resistance is often associated with the activation of bypass signalling pathways typically embedded in tumour cells or the surrounding tissue (Arteaga 2002, Wieduwilt & Moasser 2008, Cathomas *et al*. 2012, Seshacharyulu *et al*. 2012).

Inflammatory molecules have been shown to enhance EGFR oncogenic action in epithelial tumours, fostering their propensity to metastasize. Many studies on the link between inflammation and cancer have dealt with stimuli originating from the cancer microenvironment (extrinsic), providing strong support for this mechanism (Mantovani *et al*. 2008, Hanahan & Weinberg 2011, Coussens *et al*. 2013).

Microsomal PGE synthase-1 (mPGES-1), known to be induced in inflammatory as well as tumour cells by pro-inflammatory cytokines, such as interleukin-1 beta (IL-1β) and tumour necrosis factor alpha (TNFα), contributes in a critical way to tumour progression (Jakobsson *et al*. 1999, van Rees *et al*. 2003, Takeda *et al*. 2004, Kamei *et al*. 2009, Xu *et al*. 2012, Sha *et al*. 2013, Takahashi *et al*. 2014). We recently found that EGFR activation up-regulates mPGES-1, which in turn promotes phosphorylation of EGFR through prostaglandin E_2_ (PGE_2_) (Donnini *et al*. 2012).

Here we investigated whether expression of intrinsic mPGES-1 in advanced PCa cells enhanced their aggressiveness and might be critical for EGFR-mediated tumour progression. To begin to understand the mechanism underlying enhanced prostate oncogenic drive exerted by mPGES-1, we first examined human PCa specimens obtained after prostatectomy in a group of patients showing various stages of malignancy. The close association between mPGES-1 and EGFR expression observed in these tumours led us to detailed study of this connection in experiments on hormone-independent PCa cells DU145 and PC-3 *in vitro* and *in vivo*. We demonstrate that these cells have a mesenchymal phenotype and stem-like features, which are likely to confer aggressive traits. Evidence that intrinsic mPGES-1 underpins these traits is seen in cell cultures in which ablation of the mPGES-1 gene (DU145 mPGES-1^KD^) or inhibition of mPGES-1 activity prevents development of a vigorous tumorigenic phenotype. The enhanced oncogenic drive observed *in vitro* translated to nude mice *in vivo* inoculated with DU145 or PC-3 cells, as we found significantly higher tumour growth and lung metastasis formation in mice inoculated with PCa cells expressing mPGES-1. Further, blockade of EGFR *in vivo* with erlotinib indicated the possibility of quenching the oncogenic drive exerted by malignant cooperation of the two signals (PGE_2_ and EGF).

## Materials and methods

### Tumour samples

For PCa immunohistochemical study, formalin-fixed, paraffin-embedded tissue blocks from 52 radical prostatectomy specimens were retrieved from the archives of the University of Florence (Florence, Italy). Informed consent and approval according to the Helsinki Declaration were obtained from the local ethics review board. The specimens were reviewed by two genitourinary pathologists; pathological stage and tumour grade were assigned according to tumour/lympho-node/metastasis (TNM) (2010) classification and the Gleason score, respectively (Epstein *et al*. 2005, Edge *et al*. 2010). Twenty-five carcinomas were limited to the prostate (pT2, organ-confined PCa) and were moderately differentiated (Gleason score=6), whereas 27 cases (advanced PCa) were non-organ-confined tumours (pT3/pT4) with a high Gleason score (≥7) (Table 1). The median age of patients was 67.7 years (range 41–78 years).

### Immunohistochemical analysis of tumour samples

Tissue slides were deparaffinised in xylene and dehydrated in ethanol. Microwave pre-treatment in EDTA (pH 9.0) (for EGFR) or in citrate buffer (pH 6.0) (for mPGES-1 and α6-integrin) was performed for 20 min. Endogenous peroxidase activity was blocked with 3% hydrogen peroxide (v/v) for 10 min and with 3% BSA (w/v) for 30 min. The slides were incubated with primary antibodies targeting α6-integrin (1:100; Santa Cruz, Heidelberg, Germany), mPGES-1 (1:50; Thermo Scientific, Waltham, MA, USA) and EGFR (1:100; Cell Signalling, Leiden, The Netherlands) followed by chromogenic visualization using Immunoperoxidase Secondary Detection System kits (Chemicon, Billerica, MA, USA). In particular, sections were incubated for 15 min in the appropriate species-specific biotinylated secondary antibodies and then with streptavidin-conjugated HRP for 15 min. After incubation they were exposed to 3,3-diaminobenzidine tetrahydrochloride (Sigma) for 10 min to produce a brown reaction product. After counterstaining with hematoxylin, slides were washed thoroughly, dehydrated, cleared in xylene and mounted. Staining intensity was scored as negative (no staining) or positive (brown colour).

### Cell lines

DU145 WT (passages 5–20, ATCC HTB-81, certified by STRA) is a PCa cell line with high constitutive expression of mPGES-1 (Hanaka *et al*. 2009). DU145 mPGES-1 knockdown (mPGES-1^KD^ cells, passages 8–20) and non-target shRNA (mPGES-1^SC^ cells, 8–20) cells were obtained and cultured as described (Hanaka *et al*. 2009). PC-3 WT (passages 8–20, ATCC CRL-1435, certified by STRA) and LNCaP WT (passages 5–15, ATCC CRL-1740, certified by STRA) PCa cells were from ATCC. Cells were grown in RPMI (Euroclone, Pero Milano, Italy) and supplemented with 10% FBS (v/v). Human umbilical vein endothelial cells (HUVEC, passages 3–10) were from Lonza (Basel, Switzerland) (C2519A, certified by expression of CD31/105, vWFVIII, and positivity for acetylated low-density lipoprotein uptake). Cells were grown in endothelial growth medium (EGM-2) (Clonetics, Lonza) and supplemented with 10% FBS (v/v).

### Transient mPGES-1 silencing

For siRNA transfection, the siRNAs sequence (human mPGES-1: 5′-CGGGCTAAGAATGCAGACTTT-3′) was from Qiagen. The day before transfection, cells were trypsinized and 3×10^5^ cells were seeded in six-well plates. Transient transfection of siRNA was carried out using Lipofectamin 2000 (Invitrogen) according to the manufacturer's instructions. Cells were assayed 72 h after transfection.

### mPGES-1 shRNA transfection

Lenti vector plasmids for mPGES-1 knock down (Sigma) and mPGES-1+/+ (p Lenti vector with C-terminal Myc-DDK tag-NM_004878) were obtained from Sigma and Origene (Origene, Rockville, MD, USA), respectively. psPAX2 packaging plasmid (12260) and pMDG.2 envelope plasmid (12259) were obtained from Addgene (Cambridge, MA, USA).

All the plasmids were sequence-verified. To generate mPGES-1 knock down (mPGES-1^KD^) cells or mPGES-1 overexpressing (+/+) cells, 1×10^6^ HEK293 cells (Life Technologies) were transfected with 2.25 μg of PAX2 packaging plasmid, 0.75 μg of PMD2G envelope plasmid and 3 μg of pLKO.1 hairpin vector utilizing 12 μl of Lipofectamine 2000 on 10 cm plates. Polyclonal populations of transduced cells were generated by infection with 1 multiplicity of infectious units (MOI) of lentiviral particles. Three days after infection, cells were selected with 10 μg/ml puromycin (Gibco) or 20 μg/ml neomycin/kanamycin (Sigma) for 1 week.

### Epithelial-mesenchymal transition PCR array

The expression of 88 human Epithelial-mesenchymal transition (EMT) genes was profiled in DU145 cells using the EMT-RT^2^ Profiler PCR Arrays (SAAB Bioscience, Qiagen). Total RNA was isolated using an RNA Mini kit (Qiagen) and reverse transcribed using an RT-PCR kit (Qiagen). Relative expression was determined for each of the 88 genes using the formula 2^−^^Δ^^*C*^^t^.

### Real-time PCR

Total RNA was obtained using an RNA Mini kit (Qiagen). RNA (0.5 μg) was reverse transcribed using a RT-PCR kit (Bio-Rad). Premixed primers for vimentin, fibronectin, ahnak, ITGB1, Nanog, Oct4 and GAPDH (as internal control) were from Applied Biosystems. Real-time PCR was performed using SYBR Green Supermix (Bio-Rad) according to the manufacturer's instructions. RT-PCR was performed using an iCycler iQ5 PCR Detection System. The results are expressed as 2^−^^Δ^^*C*^^t^ or fold increase.

### Western blot

Tumour cells (5×10^5^) were seeded in 6 cm plates in medium with 10% fetal bovine serum (FBS) (v/v) for 96 h, then lysed and analysed. Where indicated, cells were treated with PGE_2_ (1 μmol/l), erlotinib (10 μmol/l), NS398 (10 μmol/l) or [2-(6-chloro-1*H*-phenanthro-(9,10-*d*)imidazol-2-yl)isophthalonitrile, MF63 (10 μmol/l). PGE_2_ and NS389 were from Sigma, erlotinib was from Santa Cruz and MF63 was from AbMole (Houston, TX, USA). To assess translocation of β-catenin from cytosol to nucleus, cells were trypsinized and homogenized on ice in lysis buffer containing 0.1 mmol/l EGTA, 0.1 mmol/l EDTA, 10 mmol/l Hepes, 10 mmol/l KCl, protease and phosphatase inhibitors. After incubation on ice for 15 min, Nonidet-P-40 was added to cell lysates, which were then centrifuged (3900 ***g***, 30 s). The supernatant contained the cytosolic fraction, while the pellet was solubilized in lysis buffer containing 1 mmol/l EGTA, 1 mmol/l EDTA, 20 mmol/l Hepes, 10 mmol/l NaCl, 1% protease and phosphatase inhibitors (v/v), followed by incubation on ice for 10 min and centrifuging (5480 ***g***, 5 min). The supernatant contained the nuclear fraction. An equal amount of proteins was loaded on SDS–PAGE gel and transferred to a nitrocellulose membrane. Western blot was performed as described by Donnini *et al*. (2012). Sources of antibodies were: anti-vimentin and anti-fibronectin, Sigma; anti-anhak, Abcam (Cambridge, UK); anti-β-catenin, anti-α6 integrin and anti-β1 integrin, Santa Cruz; anti-P-Tyrosine, anti-EGFR, anti-caspase3 and anti-P-ERK1/2, Cell Signalling; anti-PGE_2_ synthases, anti-COX synthases, anti-PGDH, anti-PGT and anti-EP receptors, Cayman Chemicals; anti-E-cadherin, DAKO (Milan, Italy). Images were digitalized with CHEMI DOC Quantity One software, blots were analysed in triplicate by densitometry using NIH Image 1.60B5 Software, and arbitrary densitometric units were normalized for β-actin (Sigma), tubulin (Santa Cruz) or H2A (Abcam, UK).

### Tumour growth and lung metastasis in immunodeficient mice

Experiments were performed according to Italian and EEC guidelines for animal care and welfare (EEC Law No. 86/609). The experiments were approved by the Italian Ministry of Health (215/2011-B). To assess the contribution of mPGES-1 to the anti-tumour activity of erlotinib, immunodeficient mice (5-week-old male athymic mice, Harlan, Indianapolis, IN, USA) were inoculated s.c. in the right flank with 20×10^6^ DU145 cells (mPGES-1^SC^ or mPGES-1^KD^). When tumours reached a volume of 70–100 mm^3^, the animals were randomly assigned to treatment with erlotinib (50 mg/kg, three times a week by gavage). The first treatment is reported as day 1. Serial calliper measurements of perpendicular diameters were used to calculate tumour volume in mm^3^ with the formula: shortest diameter×longest diameter×thickness of tumour in millimeter. After treatment, animals were sacrificed and tumours were collected and split in two parts. One part was immediately frozen in liquid nitrogen for western blot. The other part was embedded in Tissue-Tek O.C.T. (Sakura, Torrance, CA, USA) and frozen in liquid nitrogen for histology (Donnini *et al*. 2007). For histology, see Supplementary Data, see section on supplementary data given at the end of this article.

To assess the contribution of mPGES-1 to lung metastases, DU145 and PC-3 PCa cells (mPGES-1^SC^ or mPGES-1^KD^) were suspended in PBS at a density of 20×10^6^ cells/ml, and 250 μl of suspension was injected into the tail vein. To investigate whether EGFR inhibition affected the invasive activity of mPGES-1, cells were pre-treated with erlotinib (10 μmol/l) for 72 h before injection. After 7 weeks, immunodeficient mice were sacrificed, lungs removed and fixed in Bouin's solution and the number of metastatic colonies counted.

### MTT assay

Cell proliferation was quantified by Vybrant MTT cell proliferation assay as described (Donnini *et al*. 2007). Briefly, tumour cells (5×10^3^) were seeded in 96-multiwell plates in medium with 10% serum for 24 h and then, where indicated, exposed to erlotinib (0.1–10 μmol/l) for 96 h in 10% FBS (v/v). Results are reported as 540 nm absorbance/well.

### Adhesion

DU145 cells were maintained in 10% FBS (v/v) and then trypsinized; 5×10^4^ cells/ml in 1% FBS (v/v) medium were seeded in 96 multiwell plates coated with human fibronectin and incubated for 2 h at 37 °C. The wells were washed gently with PBS and adherent cells were fixed and stained with Diff-Quik. Adherent cells were counted by microscope in five random fields at 200×.

### Tumour-endothelium adhesion

Tumour-endothelium adhesion was performed using the CytoSelect tumour-endothelium adhesion assay kit according to the manufacturer's instructions (Cell Biolabs, San Diego, CA, USA). Briefly, HUVEC cells (10×10^5^ cells/well in 48 multiwell plates) were maintained in 10% FBS (v/v) for 48 h. After monolayer formation, DU145 cells were harvested and 1×10^6^ cells/ml were suspended in serum-free medium and incubated for 1 h in the presence of CytoTracker. Cells were washed twice and added to the endothelial cell monolayer. After 1 h cells were lysed and the fluorescence read at 480 nm/520 nm. Where indicated, endothelial cells were pre-treated for 4 h with TNFα to increase cell–cell adhesion (Sheski *et al*. 1999).

### Transendothelial migration

HUVEC cells (8×10^5^ cells/well in the filter of a 48-well transwell plate) were maintained in 10% FBS (v/v) for 48 h. After monolayer formation, DU145 cells were harvested and 1×10^6^ cells/ml were suspended in serum-free medium and incubated for 1 h in the presence of CytoTracker. Cells were washed twice with serum-free medium and 2×10^5^ cells were added to the upper side of the transwell plate. After 2 h the medium in the lower side of the plate was collected and centrifuged. Migrant tumour cells were lysed and fluorescence measured at 480 nm/520 nm.

### Cell viability assay

Tumour cells at a density of 5×10^5^ cells/ml were incubated for 24 or 48 h in medium with 0.1% FBS (v/v). After incubation, the numbers of dead cells stained with trypan blue and total cells were evaluated by optical microscope. The number of dead cells was reported as a percentage of total cells.

### Flow cytometry

Tumour cells were harvested and 3.5×10^4^ cells were incubated in ice for 30 min with primary antibody, then washed in PBS and exposed to secondary antibody for an additional 30 min. Surface CD44 and CD24 (Abcam) were quantified by flow cytometry using unlabelled monoclonal or polyclonal Ab followed by fluorescein isothiocyanate (FITC) or tetramethylrhodamine (TRIC)-labelled secondary antibodies. Cells were analysed by flow cytometry using a FACScan flow cytometer (Becton-Dickinson, Franklin Lakes, NJ, USA). Data was acquired by CellQuest and plotted using FlowJo (Tree Star, Ashland, OR, USA).

### Clonogenic assay

Tumour cells were plated in 60 mm culture dishes (1000 cells per dish) in medium containing 10% FBS. After 24 h cells were treated with erlotinib or MF63 (1–10 μmol/l) in 10% FBS (v/v) and kept in a humidified incubator for 10 days. Colonies (>50 cells) were fixed and stained with 0.05% crystal violet (w/v) (Sigma) in 10% ethanol (v/v), counted and photographed.

### Statistical analysis

Results were expressed as means±s.e.m., analysed by Student's *t*-test and/or one-way ANOVA with Bonferroni's correction. A value of *P*<0.05 was considered to denote statistical significance.

## Results

### mPGES-1 promotes the mesenchymal and stem cell-like phenotype in PCa cells

**Figure 1:**
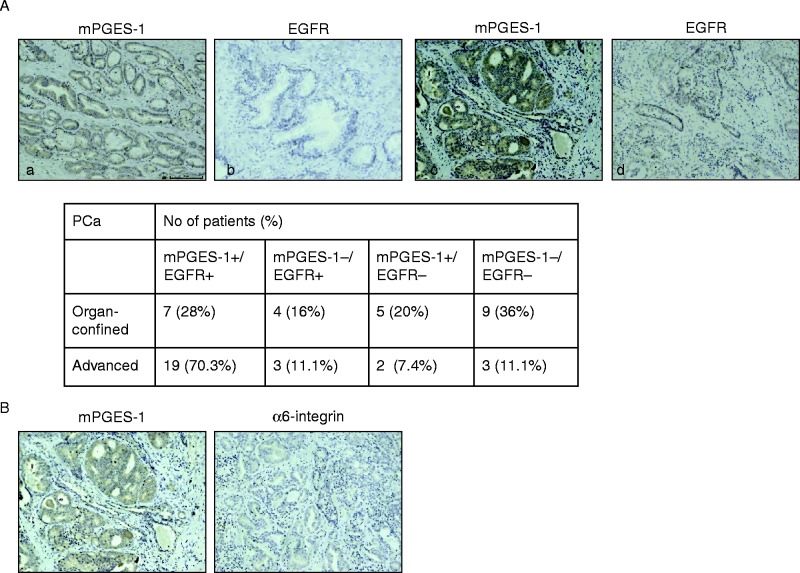
Immunohistochemical expression of mPGES-1, EGFR and α6-integrin in prostate cancer. (A) Representative images of mPGES-1 and EGFR expression in organ-confined (a and b) or advanced (c and d) prostate cancer. (B) Representative images of mPGES-1 and α6-integrin expression in advanced prostate cancer.

We investigated the correlation of mPGES-1 expression (immunohistochemistry) with staging and grading in a series of PCa cases. Overall, mPGES-1 expression was detected in 12/25 (48%) organ-confined PCa and in 21/27 (77.7%) advanced PCa (Fig. 1A). In human advanced PCa samples, both mPGES-1 and EGFR were co-expressed in a high percentage of cases (*n*=19/27, 70.3%; Fig. 1A). In the same group, α6-integrin, a stem cell marker (Marthick & Dickinson 2012, Hoogland *et al*. 2014), was negative or weakly stained (*n*=19/27; Fig. 1B). By contrast, only 7/25 (28%) of organ-confined tumours showed co-expression of mPGES-1 and EGFR (Fig. 1A, 70.3% vs 28%, see also panel a and b from an organ confined PCa sample vs c and d from an advanced PCa sample).

**Figure 2:**
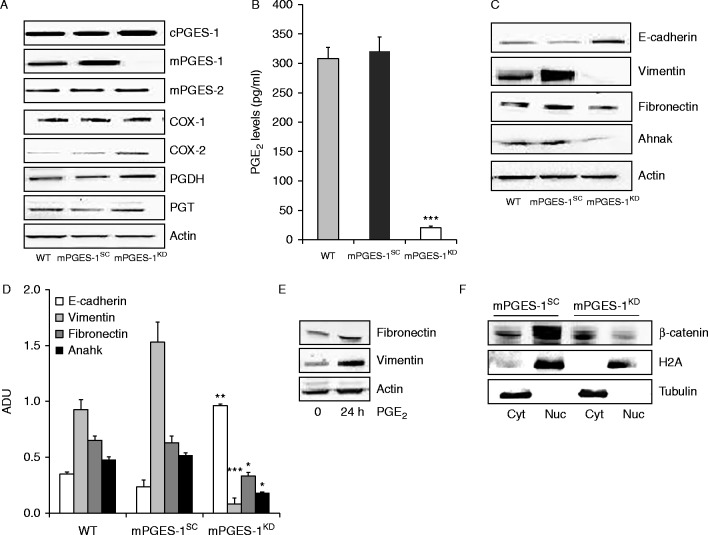
mPGES-1 promotes EMT in DU145 cells. (A) Expression of enzymes involved in arachidonic acid cascade in DU145 WT, mPGES-1^SC^ and mPGES-1^KD^ cell lines in basal condition (10% FCS) measured by western blot. (B) EIA analysis of PGE_2_ production measured in medium conditioned by DU145 cells. Results (three experiments run in duplicate) are expressed as pg/ml of PGE_2_. ***P<0.001 vs mPGES-1^SC-^. (C and D) Representative images and quantification of western blot analysis of E-cadherin, vimentin, fibronectin and ahnak expression in DU145 cells. *P<0.05, **P<0.01 and ***P<0.001 vs DU145 mPGES-1^SC^ (E) Western blot analysis of fibronectin and vimentin in mPGES-1^KD ^cells treated with PGE_2_ (1 μmol/l, 24 h). (F) Western blot analysis of β-catenin localization in DU145 mPGES-1^SC^ and mPGES-1^KD^ cells. Nuc, nucleus; Cyt, cytoplasm.

In DU145 cell line isolated from a castration-resistant human brain metastasis of PCa, characterized by mPGES-1 expression (mPGES-1^SC^) (Hanaka *et al*. 2009), the knock down for mPGES-1 (stable or transient mPGES-1^KD^) did not affect PGE_2_ receptor expression (EP1-4), while it obliterated PGE_2_ output (>90%, *P*<0.001 vs mPGES-1^SC^) (A and B, and Supplementary Figure 1, see section on supplementary data given at the end of this article). The large PGE_2_ loss occurred despite a slight increase in cyclooxygenase-2 (COX-2) expression, while other enzymes involved in PGE_2_ metabolism were either unchanged (e.g. COX-1), or only negligibly changed (e.g. the cytosolic isoform, cPGES, the microsomal type 2 isoform, mPGES-2, the prostaglandin transporter, PGT and the enzyme implicated in PGE_2_ degradation, 15-hydroxyprostaglandin dehydrogenase (PGDH)) (Fig. 2A). Compared to DU145 WT, transfection of cells with the empty vector (mPGES-1^SC^) did not affect the PGE_2_ signalling cascade (Fig. 2A). Similar results were obtained in experiments of transiently silenced mPGES-1 cells (Supplementary Figure 2A).

Because PGE_2_ regulates genes involved in EMT in tumour cell lines (Dohadwala *et al*. 2006), we assessed whether constitutively high mPGES-1 expression in DU145 affected their mesenchymal-cell-like phenotype with respect to mPGES-1^KD ^cells. The results showed that ∼1/4 of EMT-related genes (*n*=22/88) were influenced by high expression of intrinsic mPGES-1 (Fig. 2B). Notable changes occurred in transcription factors known to induce EMT, such as Snail, Slug (SNAI2 gene) and ZEB, and in several genes coding for cytoskeletal proteins, such as E-cadherin, vimentin, fibronectin and ahnak. Western blot and immunofluorescence analysis illustrated the marked loss of vimentin, fibronectin and ahnak and the increased expression of E-cadherin in stably and transiently silenced mPGES-1^KD^ cells (C and D). Reduced expression of vimentin, fibronectin and ahnak was also detected by quantitative RT-PCR (Table 1). Treatment of mPGES-1^KD ^cells with PGE_2_ (1 μmol/l, 24 h) reversed both vimentin and fibronectin expression, confirming the involvement of PGE_2_ in this process (Fig. 2E). In line with the role of PGE_2_ in cancer cell growth and E-cadherin expression (Castellone *et al*. 2005, Lu *et al*. 2012), we found massive translocation of β-catenin into the nucleus of DU145 mPGES-1^SC^ cells, while it remained localized in the cytoplasm of mPGES-1^KD ^cells (Fig. 2E and F).

**Figure 3:**
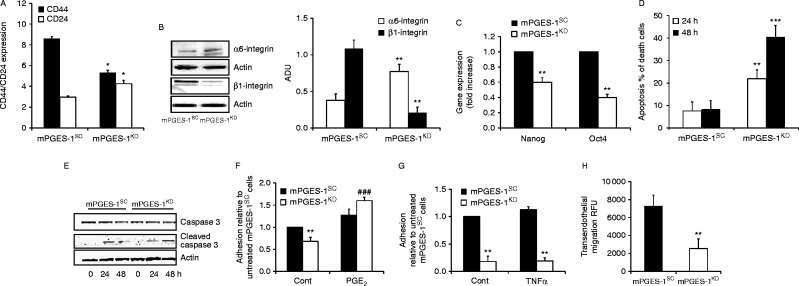
EGFR activation mediates mPGES-1/PGE_2_-dependent EMT of DU145 cells. (A) FACS analysis for CD24 and CD44 expression in DU145 mPGES-1^SC-^and mPGES-1^KD^ cells, and (B) western blot analysis and quantification of α6 and β1-integrin expression in mPGES-1^SC^ and mPGES-1^KD^ cells. Data are representative of three different experiments and quantification was performed by Image J. *P<0.01 vs DU145 mPGES-1^SC^ (C) RT-PCR analysis of Nanog and Oct4 expression in mPGES-1^SC^ and mPGES-1^KD ^DU145 cells. Data are reported as fold increase vs mPGES-1^SC ^cells. **P<0.01 vs DU145 mPGES-1^SC^. (D) Cell viability of DU145 mPGES-1^SC^ and mPGES-1^KD^ in suspension in 0.1% of serum. Results are expressed as % of dead cells. **P<0.01 and ***P<0.001 vs DU145 mPGES-1^SC^. (E) Western blot analysis of caspase 3 activation in DU145 cells grown in suspension for the indicated time. (F) Adhesion of DU145 mPGES-1^SC ^and mPGES-1^KD^ untreated or pretreated with PGE_2_ (1 μmol/l, 96 h) on fibronectin coated-96 well plate. Cell adhesion was evaluated after 2 h of incubation in 1% serum. Results (three experiments in triplicate) are expressed as % of adherent cells relative to untreated mPGES-1^SC^. **P<0.01 vs mPGES-1^SC^; ^###^*P*<0.01 vs mPGES-1^KD^. (G) CytoTracker labeled DU145 mPGES-1^SC^or mPGES-1^KD^ cells were allowed to attach to untreated or TNFα-pre-treated HUVEC monolayers in 48 well plates for 1 h. Adherent cells were lysed and quantified. Results (three experiments in duplicate) are expressed as fold increase to the adhesion of mPGES-1^SC ^on untreated HUVEC. **P<0.01 vs mPGES-1^SC^. (H) Migration of DU145 mPGES-1^SC^ and mPGES-1^KD ^toward HUVEC monolayer. CytoTracker labeled tumor cells were seeded in the upper side of 48 well-transwell and migration was evaluated after 2 h by measuring the fluorescence in the lower side of the well. Data are reported as relative fluorescence unit of three experiments run in duplicate. **P<0.01 vs DU145 mPGES-1^SC^.

We also observed a prevalence of stem-cell-like markers, regarded as indicators of tumour invasiveness, in DU145 mPGES-1^SC ^compared to mPGES-1^KD ^cells. This was indicated by the large increase in the CD44^+^/CD24^−^ ratio, the decrease in α6-integrin and the increase in β1 integrin, as well as transcription factors Nanog and Oct4 in mPGES-1^SC^ (Fig. 3A, B and C), all pluripotency maintaining factors overexpressed in PCa stem cells (Klarmann *et al*. 2009, Rentala *et al*. 2010, Marthick & Dickinson 2012, Nanta *et al*. 2013, Hoogland *et al*. 2014).Similar results were obtained for the PC-3 cell line. PC-3 cells expressed constitutive mPGES-1, and knocking down the enzyme significantlyreduced PGE_2_ output (>60%, *P*<0.01 vs mPGES-1^SC^), promoted epithelial phenotype, decreased cell clonogenicity and reduced the expression of stem cell markers (Supplementary Figure 3A, B, C, D, E and F, see section on supplementary data given at the end of this article). We also recorded anchorage-independent cell viability in mPGES-1^SC ^cells, in contrast to the drastic decline seen in mPGES-1^KD ^cells, associated with caspase-3 activation (Fig. 3D and E, and Supplementary Figure 3G and H). Adhesion studies with fibronectin coated-wells or endothelial cells revealed that mPGES-1^S^^C^ cells rapidly adhered (2 h) to the matrix or endothelium (whether or not it had been activated by TNFα to favour cell–cell interaction and transmigration), whereas mPGES-1^KD^ cells displayed a significantly delayed adhesion (F and G). Since knock down of mPGES-1 in cells does not affect cell survival *in vitro*, as measured by MTT assay (Abs 0.95±0.08 and 0.88±0.11 for DU145 mPGES-1^SC^ and mPGES-1^KD^ cells; Abs 0.74±0.06 and 0.79±0.09 for PC-3 mPGES-1^SC^ and mPGES-1^KD^ cells respectively), or cell apoptosis in suspension up to 24–48 h, we conclude that the inhibition of adhesion in mPGES-1^KD^ cells depends on the modification of cytoskeletal organization, for example of vimentin and fibronectin. Finally, by measuring trans-endothelial migration, we showed a far greater ability (nearly threefold) of mPGES-1^SC^ than mPGES-1^KD^ cells to cross cell layers (Fig. 3H), indicating the involvement of mPGES-1/PGE_2_ signalling in prostate tumour invasiveness.

### Inhibition of mPGES-1 activity suppresses stem-like phenotype

**Figure 4:**
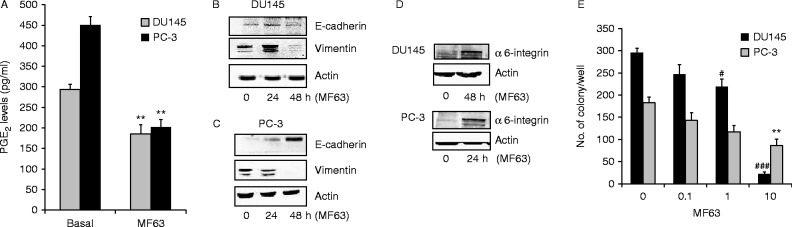
Pharmacological inhibition of mPGES-1 controls EMT markers, α6-integrin and EGFR expression, and reduces prostate cancer cells growth. (A). EIA analysis of PGE_2_ production measured in medium conditioned by DU145 or PC-3 cells after 48 h of treatment with MF63 (10 μmol/l). Results (three experiments run in duplicate) are expressed as pg/ml of PGE_2_. **P<0.01 vs basal. (B, C and D) Western blot analysis of E-cadherin, vimentin and α6-integrin expression after MF63 treatment (10 μmol/l). (E). Quantification of DU145 and PC-3 colonies in the presence or absence of MF-63 10 μmol/l. Data are reported as the number of colonies of three experiments in triplicate. *P<0.05 and **P<0.01 vs basal PC-3; ^#^*P*<0.05 and ^###^*P*<0.001 vs basal DU145.

Further evidence that the mPGES-1-PGE_2_ cascade enhances PCa cell aggressiveness was obtained by inhibiting mPGES-1 activity with the selective inhibitor MF63 (Xu *et al*. 2008). MF63 (10 μmol/l, 24–48 h) significantly inhibited PGE_2_ production and reversed the mesenchymal phenotype in DU145 and PC-3 cells, promoting E-cadherin and inhibiting vimentin expression (Fig. 4A, B and C). MF63 also upregulated α6-integrin and significantly reduced cell clonogenicity (Fig. 4D and E).

To complement the above findings, we also transfected mPGES-1 in LNCaP PCa cells, isolated from a castration-resistant human lymph node metastases lacking constitutive mPGES-1 (Supplementary Figure 4A, see section on supplementary data given at the end of this article). As expected, forced expression of mPGES-1 in LNCaP cells promoted an increase in PGE_2_ production, cell growth and development of the mesenchymal phenotype as documented by E-cadherin decrease (Supplementary Figure 4B, C, D and E).

### mPGES-1 induces growth and lung metastasis formation

In light of the observed link between mPGES-1/PGE_2_ signalling, EMT and stemness markers in PCa cells, we investigated whether the presence of mPGES-1 in DU145 and PC-3 cells influenced their growth and metastatic invasion *in vivo*.

**Figure 5:**
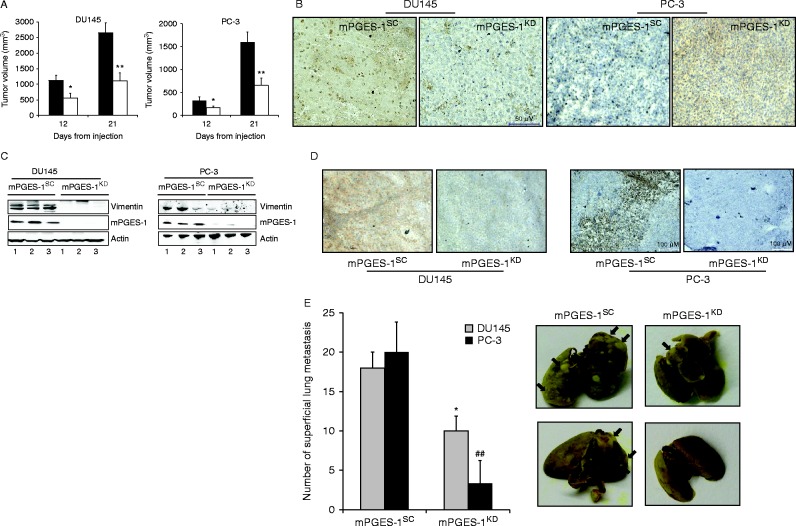
mPGES-1 expression controls *in vivo* tumor growth, vimentin and EGFR expression. (A). Tumor volume measured in athymic mice inoculated with DU145 and PC-3 mPGES-1^SC ^or mPGES-1^KD ^cells after 12 or 21 days. *P<0.05 and **P<0.01 vs mPGES-1^SC^. (B) Immunohistochemical analysis of Ki67 expression in tumor specimens derived from mPGES-1^SC^or mPGES-1^KD^. Data are representative of five tumor specimens for both tumor cell types. (C and D) Western blot and immunohistochemical analysis of vimentin expression in xenograft tumor tissues. Data are representative of five tumor specimens for both tumor cell types. (E). Quantification and images of lung metastasis after injection in mice tail vein of DU145 or PC-3 mPGES-1^SC ^or mPGES-1^KD ^cells. Images: grey frame: DU145; black frame: PC-3. *P<0.05 and ^##^*P*<0.01 vs mPGES-1^SC^

In tumours induced by inoculating nude mice with mPGES-1^SC^ or mPGES-1^KD^ cells, the volume measurements (here reported at days 12 and 21) showed significant differences, growth being 2.3-fold higher in DU145 mPGES-1^SC^ than mPGES-1^KD^ tumours, and 4.9-fold higher in PC-3 mPGES-1^SC^ than mPGES-1^KD^ tumours (Fig. 5A). Consistently, Ki-67 showed denser immunostaining in mPGES-1^SC^ than mPGES-1^KD^ tumours (Fig. 5B). Accordingly, the Ki67 score was 45±2.9% and 57±3.8% for DU145 and PC-3 mPGES-1^SC^ respectively and 21±1.9% and 29±1.8% for DU145 and PC-3 mPGES-1^KD^ respectively, indicating a higher proliferation rate in cells overexpressing mPGES-1.

Western blot (Fig. 5C) and immunohistochemistry of tumours (Fig. 5D) revealed abundant mPGES-1 and vimentin expression in mPGES-1^SC^, contrasting with the absence of these two proteins in mPGES-1^KD^ tumours.

We also investigated the contribution of mPGES-1 expression to PCa metastasis by injecting DU145 and PC-3 cells (mPGES-1^SC^ and mPGES-1^KD^ cells for both cell lines) in the tail vein of nude mice. In mice injected with DU145 or PC-3 mPGES-1^SC^, the number of lung colonies was 18±2 and 20±3.8, respectively. In contrast, knock down of mPGES-1 significantly reduced the numbers of colonies (10±1.9 and 3.3±2.8 respectively, Fig. 5E).

These results demonstrate that mPGES-1/PGE_2_ signalling plays a significant role in prostate tumour growth and metastasis development.

### mPGES-1 induces EGFR expression in prostate tumours and mediates EGFR-dependent tumour growth

**Figure 6:**
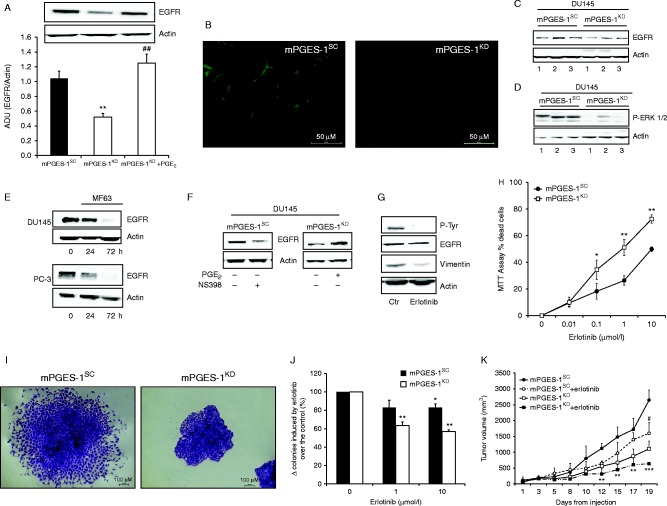
mPGES-1 expression in cells mediates prostate tumor responsiveness to erlotinib. (A) Western blot analysis of the EGFR expression in DU145 mPGES-1^SC^ and mPGES-1^KD^ cells in basal condition or treated for 96 h with 1 μmol/l PGE_2_. Graph: quantification of EGFR expression. Data (three experiments) represent the ratio between EGFR and actin. **P<0.01 vs mPGES1SC and ^##^*P*<0.01 vs mPGES-1^KD^. (B) Immunofluorescence analysis of EGFR expression in mPGES-1^SC^ and mPGES-1^KD ^cells. (C and D) EGFR expression and ERK 1/2 phosphorylation in tumor specimens derived from DU145 mPGES-1^SC^ and mPGES-1^KD^. (E and F) Western blot analysis of EGFR expression in DU145 and PC-3 mPGES-1^SC^ treated with MF63 (10 μmol/l, 24–72 h), in DU145 mPGES-1^SC^ treated with NS398 (10 μmol/l, 96 h) and in DU145 mPGES-1^KD ^treated with PGE2 (1 μmol/l, 96 h). Representative gels of three experiments. (G) Western blot analysis of EGFR phosphorylation and vimentin expression in DU145 mPGES-1^SC ^cells treated with erlotinib (10 μmol/l, 96 h). Data are representative of three experiments. (H) MTT assay of DU145 mPGES-1^S^^C^ and mPGES-1^KD^ cells treated with erlotinib (0.01–10 μmol/l, 96 h). Data (three experiments in triplicate) are reported as cell death (%). (I) Representative colonies of DU145 mPGES-1^SC^ and mPGES-1^KD^ cells. (J) Quantification of DU145 mPGES-1^SC^ and mPGES-1^KD^ colonies in the presence or absence of erlotinib 1 and 10 μmol/l. Data are reported as Δ of mPGES-1^SC^ and mPGES-1^KD^ cell colonies in the presence of erlotinib over the control (%) and are the means of three experiments in duplicate. *P<0.05 vs mPGES-1^SC^; **P<0.01 vs mPGES-1^KD^. (K) Tumor volume measured in athymic mice inoculated with DU145 mPGES-1^SC^ or mPGES-1^KD^ cells and treated with erlotinib (400 μg/mouse per three times per week, ip.). ^#^*P*<0.05, **P<0.01 and ***P<0.001 vs DU145 mPGES-1^SC^.

It is well documented that PGE_2_ favours EGF/EGFR-induced oncogenicity by directly phosphorylating EGFR (Buchanan *et al*. 2003, Donnini *et al*. 2007). However, it is unknown whether mPGES-1/PGE_2_ signalling modulates EGFR expression levels.We measured EGFR expression in DU145 mPGES-1^SC^ and mPGES-1^KD ^cells, in tumours from mice inoculated with the respective cell lines and in DU145 and PC-3 treated with MF63. We found distinctly higher EGFR expression levels in the mPGES-1^SC^ group than in the mPGES-1^KD^ group (A, B and C and Supplementary Figure 2B). Consistently, p-ERK1/2, a known downstream effector of EGFR signalling, was higher in mPGES-1^SC^ than mPGES-1^KD ^tumours (Fig. 6D), suggesting that EGFR signalling plays a role in mPGES-1 enhancement of tumour growth.

Moreover, treatment of DU145 and PC-3 mPGES-1^SC ^cells with mPGES-1 inhibitor MF63 (10 μmol/l) and DU145 mPGES1^SC^ cells with COX-2 inhibitor NS398 (10 μmol/l, 96 h) inhibited EGFR expression (Fig. 6E and F), while exogenous PGE_2_ (1 μmol/l, 96 h) increased EGFR protein levels in DU145 mPGES-1^KD^ cells, supporting involvement of PGE_2_ in promoting EGFR over-expression in prostate tumours.

Further evidence of involvement of mPGES-1/PGE2 signalling in EGFR expression was obtained in experiments *in vitro* and *in vivo* using erlotinib, a known inhibitor of EGFR. *In vitro*, erlotinib administration to DU145 cells (10 μmol/l) abrogated EGFR phosphorylation and downregulated vimentin expression (Fig. 6G). Phosphorylation of EGFR was independent of EGF expression, which was affected in mPGES-1^KD^ cells but not in mPGES-1^SC^ (Supplementary Figure 5, see section on supplementary data given at the end of this article). Erlotinib also reduced cell viability in DU145 mPGES-1^KD^ cells and functionally halved the number of cell colonies (number of colonies: mPGES-1^SC^=58±4 vs mPGES-1^KD^=27±5) (Fig. 6H, I, J, K and L). *In vivo*, erlotinib decreased tumour growth in DU145 mPGES-1^SC^ and mPGES-1^KD^-bearing mice with respect to the vehicle-treated group (area under curve: mPGES-1^SC^+erlotinib=12360 vs mPGES-1^KD^+erlotinib=5664, Fig. 6M). Erlotinib treatment was more effective in reducing tumour volume when mPGES-1 was knocked down (Fig. 6I), but it did not affect the number of metastases for DU145 and PC-3 cell lines (number of metastases: mPGES-1^SC^=20±4.8 and mPGES-1^KD^=7.5±3.5 for DU145; mPGES-1^SC^=17. 7±4.2 and mPGES-1^KD^=5.3±3.4 for PC-3).

All together, this data provides clear evidence of the role played by the mPGES-1/PGE_2_ pathway in inducing a mesenchymal phenotype and stemness in PCa cells, thus reinforcing EGFR tumorigenic drive.

## Discussion

The present study shows that by eliciting mesenchymal and stem-cell-like traits and EGFR expression, the tumour intrinsic inflammatory mPGES-1/PGE_2_ pathway cooperates with the EGFR oncogene to promote an aggressive PCa phenotype.

As an experimental paradigm we used DU145 and PC-3 cells in which mPGES-1 was stably or transiently knocked down by mRNA silencing (mPGES-1^KD^), comparing them with prostate cells containing a negative control non-targeting shRNA plasmid (mPGES-1^SC^). Further, evidence of the specificity of mPGES-1/PGE_2_ signalling in PCa aggressiveness was obtained by pharmacological inhibition of the enzyme with the selective MF63 inhibitor (Xu *et al*. 2008).

Inflammation plays a role in the development and progression of many cancers, including PCa, and multiple pro-inflammatory molecules are associated with PCa recurrence (Ørsted & Bojesen 2013). Here we investigated the contribution of the mPGES-1/PGE_2_ pathway to EMT, a process that promotes acquisition of mesenchymal traits, such as enhanced growth and migratory capacity, invasiveness and resistance to apoptosis, by epithelial cells. We observed that mPGES-1 knockdown influenced a set of genes promoting EMT in tumour cells, such as genes coding for transcriptional activity (Snail, Slug and ZEB), which were significantly downregulated. The effect on the cytoskeletal protein vimentin, which became undetectable in mPGES-1^KD^ and in cells treated with MF63, was particularly striking. A number of other changes occurred in mPGES-1^KD^ cells. They included downregulation of insulin growth factor binding protein-4 (IGFBP-4) and the integrin β1 (ITGB1) gene, re-localization of β-catenin and overexpression of E-cadherin. All these markers are known to be associated with PCa aggressiveness (Damon *et al*. 1998, Miyake *et al*. 2000, Mohan & Baylink 2002, Bijnsdorp *et al*. 2013, Carbonell *et al*. 2013). Regulation of these markers may therefore be causally related to the loss of function parameters observed in mPGES-1^KD^ cells.

mPGES-1 expression in PCa cells was clearly associated with stem-like features as demonstrated by the greater survival ability of mPGES-1^SC^ cells in suspension compared to mPGES-1^KD^ cells. Additional evidence of mPGES-1-linked stemness was a significant shift in the CD44/CD24 ratio, decreased β1-integrin, increased α6-integrin expression and increased transcription factors Nanog and Oct4 (Nanta *et al*. 2013). Collectively, these results provide evidence of involvement of mPGES-1/PGE_2_ in promoting EMT and stemness in mPGES-1^SC^ tumour cells, since its ablation and inhibition impairs their inherent potential to survive in suspension and to transmigrate to endothelial cells.

EGFR studies provided another means to examine the interplay between PGE_2_ input and oncogenic drive mediated by the EGFR system. The picture emerging is one of reciprocal activation producing vigorous PCa progression when both components (PGE_2_ and EGF) of this circuit are maximally expressed, as in mPGES-1^SC^ cells, and conversely, a reduced outcome in mPGES-1^KD^ and in mPGES-1-inhibited cells, in which both components are downregulated. This circuit appears to be operant in the clinical setting here reported, as in human prostate tumours mPGES-1 and EGFR are concomitantly expressed in specimens with a high Gleason score compared to specimens of organ-confined lesions with a low Gleason score. Thus, mPGES-1/PGE_2_ signalling empowers tumour cells to disseminate and seed metastases by activating EMT and stemness in PCa.

The present findings illustrate the role of mPGES-1 signalling in influencing EGFR-mediated oncogenicity and in contributing, in association with the EGFR pathway, to aggressiveness in PCa. Indeed, erlotinib treatment reduced tumour development in the mPGES-1^SC^ group to approximately the same level as that observed in mPGES-1^KD^ tumours. Further, ablation of PGE_2_ production significantly reduced lung metastasis in mice, indicating that mPGES-1 signalling plays a key role in EMT and stemness of PCa cells (Oskarsson *et al*. 2014). The combination of mPGES-1 knockdown or pharmacological inhibition of mPGES-1 by MF63 with erlotinib results in a more effective strategy to inhibit PCa cell growth. Ability to undergo EMT and acquire stem-cell-like features enables tumours to expand, spread and resist chemotherapy/target therapy (Clevers 2011, Hoggatt *et al*. 2013). PGE_2_ release in the tumour microenvironment was recently shown to be responsible for tumour initiation and repopulation, hence inhibitors of PGE_2_ production interfere with tumour progression and chemoresistance (Kurtova *et al*. 2015). Our study demonstrates that mPGES-1 ablation or inhibition in human PCa cells suppresses their overall oncogenic drive and reduces their stemness and invasiveness. We conclude that the mPGES-1 gene may be considered a signature gene for identifying a subtype of rapidly progressing prostate tumours, which, in the case of overexpression of EGFR, may benefit from combined treatment with inhibitors of EGFR, tyrosine kinase and PGE_2_.

## Supplementary data

This is linked to the online version of the paper at http://dx.doi.org/10.1530/ERC-15-0277.

## Figures and Tables

**Table 1:** Gene expression of vimentin, fibronectin and ahnak in DU145 cells expressing mPGES-1

**Gene**	**Mean 2^−(^^Δ^^*C*^^t)^**	**s.d.**
Vimentin	12.1	2
Fibronectin	2	0.1
Ahnak	1.6	0.2
